# The Relationships Amongst Pediatric Nurses' Work Environments, Work Attitudes, and Experiences of Burnout

**DOI:** 10.3389/fped.2021.807245

**Published:** 2021-12-21

**Authors:** Laura Buckley, Whitney Berta, Kristin Cleverley, Kimberley Widger

**Affiliations:** ^1^Lawrence S. Bloomberg Faculty of Nursing, University of Toronto, Toronto, ON, Canada; ^2^Hospital for Sick Children, Toronto, ON, Canada; ^3^Institute for Health Policy, Management and Evaluation, University of Toronto, Toronto, ON, Canada; ^4^Margaret and Wallace McCain Center for Child, Youth and Family Mental Health, Centre for Addiction and Mental Health, Toronto, ON, Canada

**Keywords:** nurses, pediatrics, burnout—professional, organizational behavior (OB), critical care

## Abstract

**Background:** Pediatric nurses care for some of the most vulnerable patients in our healthcare system. Research on health care provider organizational behavior shows that the quality of care nurses provide is directly related to their well-being, influenced by Burnout and job stress, in the workplace. However, most of the research conducted on nursing populations neglects to separately study nurses who care for children. In a resource limited system where health care provider well-being is recognized as a priority, it is important for administrators to understand the environmental and attitudinal work factors most influential to pediatric nurse work outcomes in order to target optimization strategies. The aim of the study was to identify which *modifiable* work environment factors, e.g., [Incivility, Perceived Organizational Support, Quality of Work-life] make the *greatest* contribution to the work outcome of Burnout (i.e., Personal Accomplishment, Emotional Exhaustion, Depersonalization) in pediatric nurses.

**Methods:** A cross-sectional survey design was used at a large quaternary care pediatric hospital in Toronto, Canada. We administered a survey to a convenience sample of all registered nurses with >3 months experience in the Pediatric, Cardiac, and Neonatal Intensive Care Units from January 2021–March 2021. Path analysis was used to test our proposed model which was specified *a priori* based on a review of the literature.

**Results:** 143 nurses completed the survey. Path analysis of the tested model resulted in good fit. Quality of Work-life had the largest direct effect on Work Engagement (β = 0.582, S.E. = 0.111, *p* < 0.001). Work Engagement had the largest direct effect on Personal Accomplishment (β = 0.68, S.E. = 0.53, *p* < 0.001). Quality of Work-life had the largest indirect effect on Personal Accomplishment (β = 0.4, S.E. = 0.65, *p* < 0.001), Emotional Exhaustion (β = −0.33, S.E. = 0.87, *p* < 0.001), and Depersonalization (β =−0.17, S.E. = 0.41, *p* = 0.006), respectively. Work Engagement had the largest total effect on Personal Accomplishment (β = 0.68, S.E. = 0.64, *p* < 0.001) and the third largest total effect on Emotional Exhaustion (β = −0.57, S.E. = 0.83, *p* < 0.001). Quality of Work-life had the second largest total effect on Work Engagement (β = 0.58, S.E. = 0.11, *p* < 0.001) indicating that Quality of Work-life is mediated through Work Engagement for its effect on Burnout.

**Conclusions:** Our results indicate work environment and work attitude factors that can provide organizational leadership with a targeted focus to reduce pediatric critical care nurse Burnout, and thus improve provider well-being, in a resource limited system.

## Background

Pediatric nurses care for some of the most vulnerable patients in our healthcare system. These nurses skillfully manage the highly specialized care of children and the complex family dynamics that are inherent to the work (1). Pediatric nurse well-being in the workplace has been shown to be directly and positively related to nurses' attitudes about engaging with patients and families (2), and the quality of care provided (3–6). Pediatric nurses are a separate population from nurses who care for adults because of the specialized nature of providing care to children. Children are typically seen as a vulnerable population, and along with this, there is a high potential for empathetic engagement and inherent complexities in the relationships with families (7, 8). More specifically, pediatric/neonatal critical care nurses care for the most severely ill and injured children at the highest risk of death (9). As the stakes for this patient population are arguably the highest in the hospital, stressors of the work environment are enhanced; the care needs are highly complex and the stress to the families adds additional challenges (10, 11). Pediatric/neonatal critical care nurses are a subspecialty within a specialty. A supply-demand issue ensues as these nurses cannot be easily replaced or supplemented. Thus they are continually asked to do more (care for more patients, run more technology) with less (less time, resources, support) (1). Much of the organizational behavior research conducted to date on nursing populations has focused on general adult care nurses and excluded studying nurses who care for children (12, 13), particularly in pediatric critical care settings.

Work outcomes refer to occupational performance factors that are influenced by work attitudes and the work environment (14). The current study focuses on the work outcome of Burnout as it is one of the most established organizational behavior concepts, with over 40 years of literature available on the topic across numerous industries (15). Maslach and Jackson (1981) define Burnout by three components; Emotional Exhaustion, Depersonalization, and lack of Personal Accomplishment (16). Emotional Exhaustion refers to nurses feeling emotionally drained from their work; Depersonalization is the development of cynicism, particularly toward patients; and lack of Personal Accomplishment refers to nurses' feelings of dissatisfaction with the care they are providing (16). Nurse Burnout impacts at the level of the provider, the patient, and the organization (17). Burnout is positively associated with nurses' intent to leave their jobs (18), decreased quality of life (19), and negatively associated with the safety of the work environment (3, 4).

Nurses working in critical care commonly experience Burnout, with rates as high as 73% for Emotional Exhaustion, 60% for a lack of Personal Accomplishment, and 48% noting Depersonalization (20). Most of the currently available literature on pediatric nurse work outcomes, such as Burnout, focus on factors like race, marital status, or the experience of death in the workplace (21–23) or on high cost/low yield factors like nurses' personality traits (22). The former set of factors are non-modifiable and cannot be feasibly changed (e.g., race), while high cost/low yield factors are technically modifiable but it would not be fiscally or temporally responsible to try and impact (e.g., personality traits) (24). So while it is beneficial to be aware of the impact of these factors, they are not ideal targets for efficient modification by health care organizations. In a health care climate where resources are limited, it is important for administrators to know which environmental and attitudinal work factors make the greatest contribution to pediatric nurse work outcomes to target their optimization strategies in the most cost-effective way.

**Figure 1:**
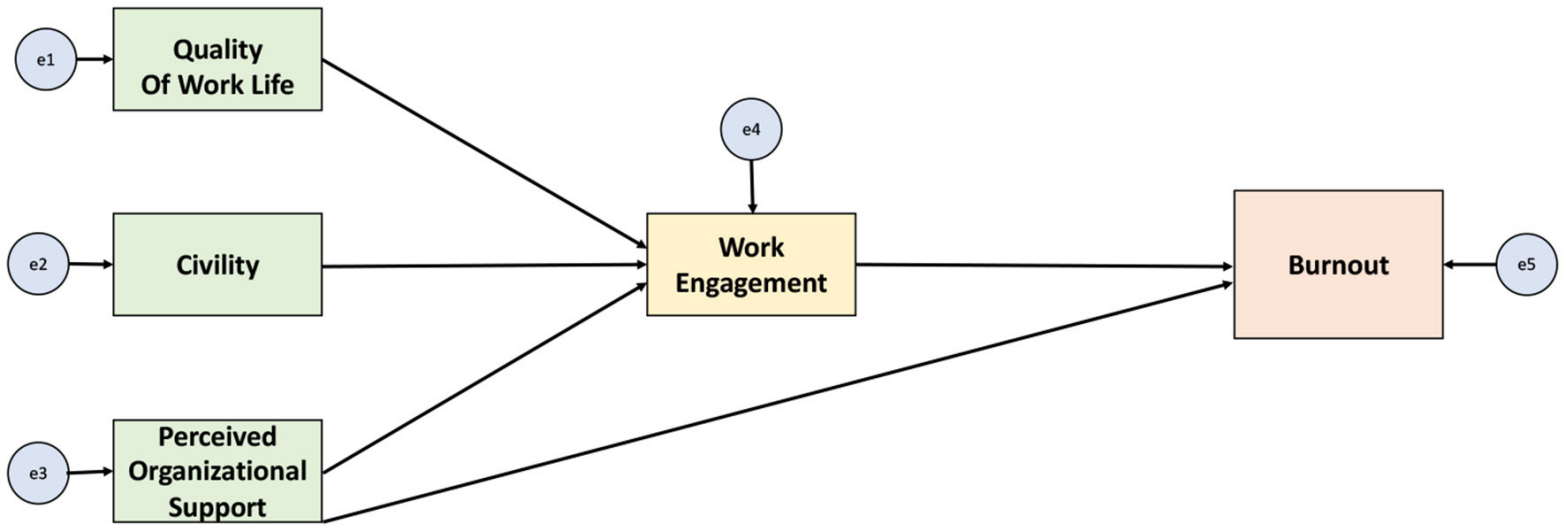
The relationship between factors of the work environment, work attitudes, and the work outcomes of Burnout.

Our study began with a review of the literature on what is known about pediatric nurse Burnout (17). From there, an additional search of the literature was undertaken to investigate factors that impact Burnout in the broader health care population. Using this data and the framework of the Theory of Reasoned Action we proposed a conceptual model. Figure 1 illustrates the proposed conceptual model where the pediatric nursing work environment influences work outcomes through work attitudes, thus influencing work outcomes directly.

### Factors Related to Burnout

#### Work Environment

##### Incivility in the Workplace

Workplace Incivility is defined as; “low intensity behaviors that are rude, lack consideration of others, in violation of workplace norms for respect, where the intent to harm is ambiguous” (25). These behaviors serve as a pre-cursor for an exchange, or spiral, of coercive behavior.

##### Quality of Work Life

Sirgy et al. define quality of work life (QWL) as; “employee satisfaction with a variety of needs through resources, activities, and outcomes stemming from participation in the workplace. Thus, satisfaction from workplace experiences contributes to job satisfaction and satisfaction in other life domains. Satisfaction in the major life domains (e.g., work life, family life, home life, leisure life) contributes directly to satisfaction with overall life” (26).

##### Perceived Organizational Support

Perceived organizational support (POS) is defined by the extent to which the employee perceives “the organization values their contributions and cares about their well-being” (27). POS is inversely related to nurse Burnout (27–29); POS is positively related to Work Engagement in nurses (30); and POS is inversely related to nurses' intent to leave (31–33).

#### Work Attitudes

##### Work Engagement

Schaufeli, Bakker, & Salanova (2006) define Work Engagement as; “a positive, fulfilling work-related state of mind that is characterized by vigor, dedication, and absorption. To be able to increase nurses' engagement with their patients and families we must think about possible interventions in the same light. Highly engaged nurses are essential for ethical, safe, and comprehensive care (34). Work Engagement may be challenging to directly modify, but work outcomes can be influenced *through* more easily modifiable factors of the work environment (14). Similar to work environment factors, pediatric nurses have not been separately studied in the nursing Work Engagement literature.

### Study Objectives

**1)** To test a model of modifiable work environment features and attitudes in relation to the work-related outcome of Burnout in a sample of pediatric critical care nurses.
**2)** To rank the modifiable work environment factors based on their relationship with Burnout among pediatric critical care nurses.

## Methods

This study used a cross-sectional survey design to test a model of the relationships between organizational and attitudinal factors and Burnout in a convenience sample of nurses at a large quaternary care pediatric hospital in Toronto, Canada. The study was approved by the Research Ethics Board (#1000072502) at the Hospital for Sick Children.

### Study Setting

The study took place in a 300-bed tertiary care hospital with a 41-bed critical care unit and a 38-bed neonatal intensive care unit in Toronto, Canada. A total of 443 Registered Nurses (RNs) work in the Cardiac Critical Care Unit (CCCU), Pediatric Critical Care Unit (PICU) and the Neonatal Intensive Care Unit (NICU) combined.

### Sample and Recruitment

Inclusion criteria required that nurses had worked in the PICU, CCCU, or NICU for >3 months. Nurses undergoing orientation were excluded. All nurses in this organization are Registered Nurses (RNs). Nurses on leave (medical, parental, or otherwise) were excluded from participation as they were not actively working on the unit and not contactable via the hospital email server. All nurses working on these units were contacted via email for participation in the study. QR codes linking to the survey were also advertised on posters throughout the units. Surveys were completed and submitted automatically and anonymously online through *REDCap Software* (35). Data collection was conducted from January 6, 2021 to March 22, 2021. Of note, this data collection period was during the Coronavirus disease 2019 (COVID-19) pandemic. For context, participants were asked if they have cared for a COVID-19 positive patient or patient under investigation for COVID-19. Participant consent was implied by survey submission. Participants were offered a $5 coffee card as a thank you for their participation.

### Data Collection Tools

The survey was made up of established instruments, previously used with nurses, that had good psychometric properties. Demographic information was also collected, along with a final open-ended question for nurses to include any other thoughts on the topic.

#### Maslach Burnout Inventory

Burnout was measured using the Maslach Burnout Inventory for Human Services Survey for Medical Personnel [MBI-HSS(MP)] with subscales for Depersonalization, Emotional Exhaustion, and Personal Accomplishment. Each subscale's items are scored on a Likert scale from 0 to 6, indicating the frequency that the item applies in the providers experience ranging from with 0 indicating “never” to 6 indicating a frequency of “everyday” (36).

#### Work Attitudes

##### Utrecht Work Engagement Scale

Work Engagement was measured by the Utrecht Work Engagement Scale shortened 9-item version (UWES-9). [All items are measured on a 7-point Likert scale (0 = never–6 = always) and Work Engagement scoring categories include “very high”, “high”, “average”, “low” and “very low” (37)].

#### Work Environment Features

##### Workplace Incivility Scale

The Workplace Incivility Scale (WIS), created by Cortina et al. was selected because it is a 7-item tool and measures a single construct of Workplace Incivility with an Cronbach's alpha = 0.89 and demonstrated validity (38). The tool is scored on a 7-point Likert scale where respondents self-report how often they experience instances of Workplace Incivility on a scale from 0 = never to 6 = daily (38).

#### The Survey of Perceived Organizational Support

The Survey of Perceived Organizational Support shortened 8-item tool uses a 7-point Likert scale (1 = strongly disagree to 7 = strongly agree) (39).

#### Quality of Work Life Measure

The Quality of Work Life Measure, developed by Sirgy et al. in 2001, combines both needs satisfaction and spillover theories within the 7-factor, 16 item tool with response options on a 7-point Likert scale (1 = very untrue to 7 = very true) (26).

Where multiple options or versions of tools were available, we selected the shortest version if there were similar psychometric properties to reduce the overall survey length. (Cronbach's alpha was calculated for each data collection tool; all had acceptable reliability with a Cronbach's alpha >0.7 Data collection tools and their psychometric properties for this sample are in Table 1).

**Table 1:** Data collection tools and psychometric properties.

**Factor**	**Tool**	**Number of questions**	**Scale**	**Cronbach's alpha**	**Scoring**	**Our cronbach's** **Alpha**
**Civility**	The workplace incivilityscale	7	Likert (0–6)	0.93	Total mean across items	0.8854
**Perceived organizational support**	Survey of perceived organizational support—shorted version	8	Likert (1–7)	0.86–0.88	Total means across items	0.8110
**Quality of work life**	Quality of work life measure	17	Likert (1–7)	0.85	Total means across items	0.8622
**Work engagement**	Utrecht work engagement scale-9	9	Likert (0–6)	0.89–0.97	Total Mean across items	0.8516
**Burnout**	Maslach burnout inventory –HSS	22	Likert (0–7)	0.89	Three subscale scores (EE, DP, PA)	0.8661

#### Analysis

Sample demographics and scale scores were summarized using descriptive statistics including means, standard deviations (SD), counts, and proportions as appropriate for the type of data and scoring guidelines for the scales. In addition, a correlation matrix was estimated to determine the relationship between each of the variables used in the path model.

##### Analysis Objective 1

Path analysis was used to test the model (Figure 1) of the relationships amongst *modifiable* work environment features, work attitudes and Burnout among pediatric critical care nurses. Path analysis is a component of Structural Equation Modeling (SEM); a simple case that does not include latent variables. Path analysis is most appropriate for our study as our model does not contain latent variables, therefore no measurement model is needed. **Three** subscales of Burnout were included in the model: Emotional Exhaustion, Depersonalization, Personal Accomplishment. Quality of Work-life, Perceived Organizational Support and Civility were the exogeneous variables, and Work Engagement was modeled to mediate the relationship between the exogenous variables and the outcome. In addition, a path for the direct effect of Perceived Organizational Support on Burnout was tested (Figure 1). STATA (Version 15) was used to conduct the path analysis and effect sizes were calculated (40). Indirect effects were calculated using bootstrapping. Acceptable model fit was indicated by a non-significant χ^2^ value, a comparative fit index (CDI) >0.90, a Tucker-Lewis index (TLI) 0.0.90, a root mean square standard error of approximation (RMSEA) <0.05 (41). Missing values were addressed using full information maximum likelihood estimation (FIML).

##### Objective 2

Modifiable work environment factors were ranked (by their correlation coefficient) based on their contribution to explaining Burnout among pediatric critical care nurses (42).

##### Sample Size

Minimum sample size for our study was calculated using the N:q rule, there were *q* = 7 parameters that require estimates. The ratio of 10:1 was used, indicating a minimum sample size of *n* = 70 (41). In order to improve the trustworthiness of the results, we chose to use a ratio of 15:1, for a minimum sample size of *n* = 105 in order to adequately power the analysis.

## Results

### Response Rate

The survey link was distributed to 443 nurses in the PICU/NICU/CCCU. The distribution of respondents was 44.8% from PICU, 37.1% from NICU, and 17.5% from CCCU. Of the 158 surveys opened, 15 had no data thus were excluded, and 143 were fully or partially completed for a response rate of 32.3%. Surveys that had any complete instruments were used in the calculation of mean scores. Only surveys that had all instruments completed were used for the path model (*n* = 117). Surveys with missing data were analyzed for any commonalities. Distributions for years of experience, FTE, and highest degree achieved were all similar distribution to the fully completed survey sample. NICU incomplete surveys were slightly higher amongst the incomplete surveys, perhaps indicating a higher level of interruptions during completion. At baseline, NICU nurses carry a higher patient load (more 2:1 assignments) than the other two units.

### Demographic Characteristics

The majority of respondents worked full time (>0.8 Full-time equivalent) and completed a bachelor's degree as their highest degree held. Our sample was fairly evenly distributed by nurses of different years of experience. The majority of our sample had also taken care of a COVID-19 positive patient (Table 2).

**Table 2:** Respondent characteristics (*n* = 143).

**Respondent characteristics**	***N* (%)**
**Unit**	
PICU	64 (44.8%)
CCCU	53 (37.1%)
NICU	25 (17.5%)
Prefer not to respond	1 (0.7%)
**Years of work experience**	
0–5 years	45 (31.5%)
6–10 years	42 (29.4%)
>10 years	56 (39.2%)
**Full time equivalents**	
<0.5	3 (2.0%)
0.5–0.8	26 (18.2%)
>0.8	56 (39.2%)
Prefer not to respond	4 (2.8%)
**Highest academic degree achieved**	
Diploma	6 (4.2%)
Bachelor's Degree	120 (84%)
Master's Degree	17 (11.9%)
**Cared for a COVID-19 patient under investigation or positive patient?**	
Yes	124 (86.7%)
No	19 (13.3%)

*Pediatric Intensive Care Unit (PICU), Cardiac Critical Care Unit (CCCU), Neonatal Intensive Care Unit (NICU)*.

A summary of each of the mean scores for each of the tools used in the path analysis can be found in Table 3. The mean Emotional Exhaustion score was 24.6 with 40% scoring high level of Emotional Exhaustion. The mean Depersonalization score was 9.1 with 44.6% scoring a high level of Depersonalization. The mean Personal Accomplishment score is 32.8 with 47.7% scoring a high level of Personal Accomplishment (Table 4). The correlations between Work Engagement, Quality of Work-life, Workplace Incivility, Emotional Exhaustion, Depersonalization, and Personal Accomplishment were all significant (Table 5).

**Table 3:** Summary data of Work Environment and Work Engagement scores.

**Variable**	**Obs**.	**Mean (SD)**	**Min**.	**Max**.
**Quality of work-life**	130	4.83 (0.81)	2.44	6.44
**Perceived organizational support**	127	3.12 (0.82)	1	4.50
**Work engagement**	124	3.92 (0.82)	1.89	5.67
**Workplace incivility**	124	2.34 (0.80)	1	4.86

**Table 4:** Burnout subscale scores by category.

**Emotional exhaustion** **(0–54)**	***n =* 130**	**Depersonalization** **(0–30)**	***n =* 130**	**Personal accomplishment** **(0–48)**	***n =* 130**
**High (≥27)**	52 (40%)	High (≥10)	58(44.6%)	High (0–33)	62 (47.7%)
**Moderate (19–26)**	49 (37.7%)	Moderate (6–9)	38 (29.2%)	Moderate (34–39)	50 (38.5%)
**Low (0–18)**	29 (22.3%)	Low (0–5)	34 (26.2%)	Low (≥40)	18 (14%)

**Table 5:** Number of respondents, Pearson correlations, scale means and standard deviations *(n* = *117)*.

**Study variables**	**Mean (SD)**	**1**	**2**	**3**	**4**	**5**	**6**	**7**
**1. Quality of Work Life**	4.7 (0.80)	1.0						
**2. Perceived organizational support**	3.2 (0.78)	0.57^**^	1.0					
**3. Workplace incivility**	2.32 (0.80)	−0.49^**^	0.3^**^	1.0				
**4. Work engagement**	3.92 (0.83)	0.53^**^	0.28^**^	−0.21^*^	1.0			
**5. Burnout: emotional exhaustion**	24.6 (9.49)	−0.52^**^	−0.37^**^	0.32^**^	−0.63^**^	1.0		
**6. Burnout: depersonalization**	9.10 (5.35)	−0.31^**^	−0.28^**^	0.19^*^	−0.33^**^	0.5^**^	1.0	
**7. Burnout: personal accomplishment**	33.03 (5.59)	0.49^**^	0.19^*^	−0.19^*^	0.65^**^	−0.38^**^	−0.27^**^	1.0

* *p < 0.5*,

** *p < 0.01*.

#### Objective 1: Results of Path Analysis

**Figure 2:**
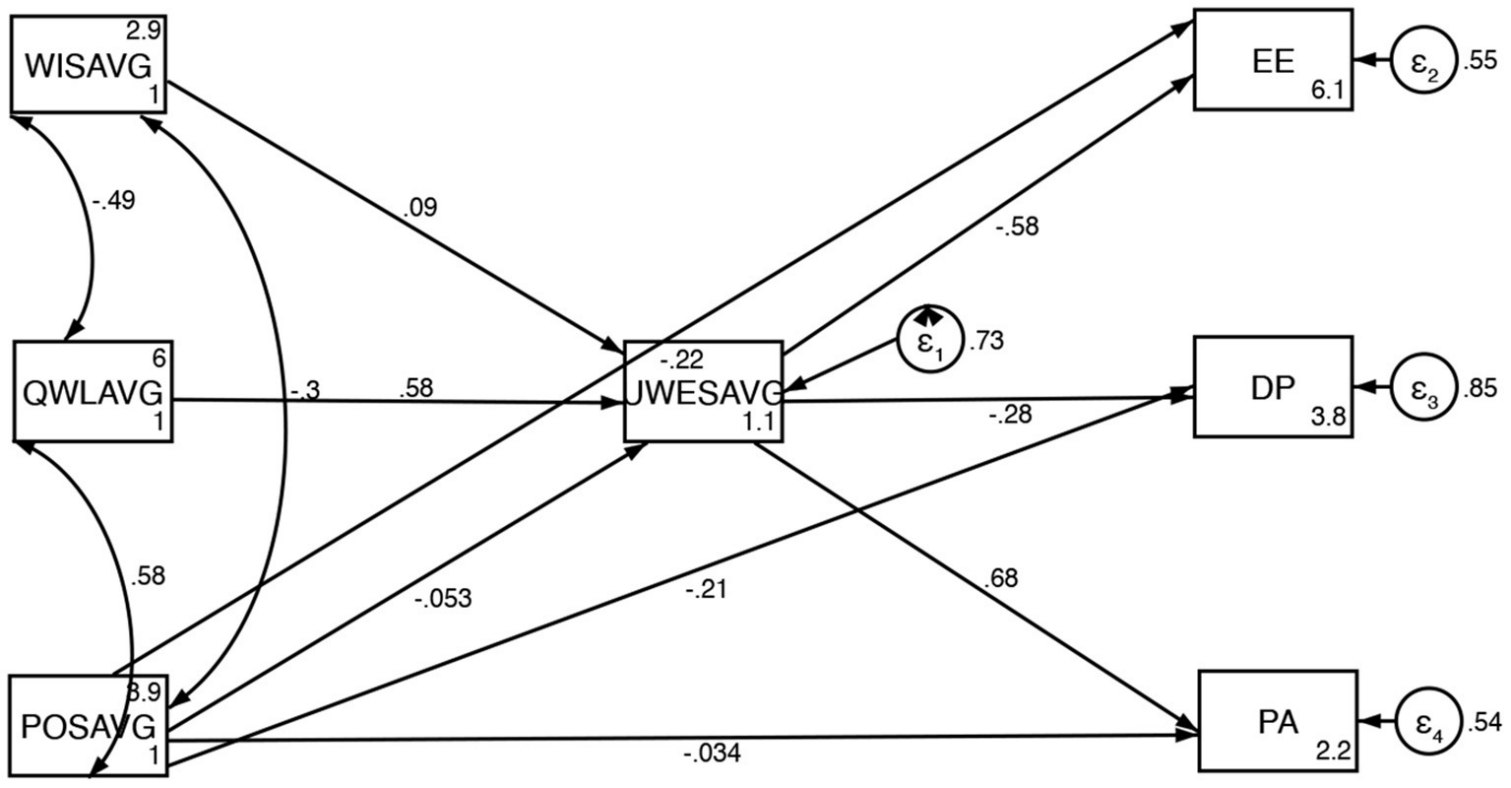
Conceptualized framework with standard coefficients from path analysis. WISAVG, Workplace IncivilityScore Average; QWLAVG, Quality of Work-life Average; POSAVG, Perceived Organizational Support Average; UWESAVG, Utrecht Workplace Engagement Survey Average; EE, Emotional Exhaustion; DP, Depersonalization; PA, Personal Accomplishment.

Path analysis of the tested model resulted in good fit, as demonstrated by a non-significant (χ^2^(6) = 10.6, *p* = 0.1015), Root mean squared error of approximation (RMSEA) = 0.08, Comparative Fit Index (CFI) = 0.90, Tucker Lewis Index (TLI) 0.93, and CD = 0.33. Our model accounts for 27% of the variance in Work Engagement scores, 44% of the variance in Emotional Exhaustion scores, 16% of the variance in Depersonalization scores, and 46% of the variance in Personal Accomplishment scores. The coefficient of determination for the entire model is low (CD = 0.33) which is common for social science based research (43). Figure 2 presents the significant standardized coefficients from the path analysis.

##### Work Outcomes

Emotional Exhaustion is strongly inversely associated with Work Engagement (β = −0.570, *p* < 0.001) and moderately inversely associated with Perceived Organizational Support (β = −0.226, *p* = 0.003). Depersonalization is moderately inversely associated with Work Engagement (β = −0.290, *p* < 0.001) and Perceived Organizational Support (β = −0.200, *p* = 0.028) Personal Accomplishment is strongly associated with Work Engagement (0.680, *p* < 0.001) and not statistically significantly associated with Perceived Organizational Support (β = −0.034, *p* = 0.668). The subcomponents of Burnout are weakly associated with each other (Figure 2).

##### Work Environment

Workplace Incivility is not associated with Work Engagement (β = 0.090, *p* = 0.333). Quality of Work-life is strongly positively associated with Work Engagement (β = 0.580, *p* < 0.001) Perceived Organizational Support is not associated with Work Engagement (β = −0.053, *p* = 0.593) (Figure 2).

##### Direct Effects of Variables on Burnout

Quality of Work-life had a statistically significant direct positive association with Work Engagement. Both Work Engagement and Perceived Organizational support had significant direct effect on Emotional Exhaustion and Depersonalization. Work Engagement had a significant direct effect on Personal Accomplishment (Table 6).

**Table 6:** Direct effects with standardized coefficients.

	**Coefficient**	**Standard Error (S.E.)**	***z***	***P* > |*z*|**	**Standardized coefficient (β)**	**Rank**
**Work engagement**						
Workplace incivility	0.095	0.098	0.97	0.333	0.09	
Quality of work-life	0.608	0.111	5.48	0.000	0.582*	**2**
Perceived organizational support	−0.055	0.103	−0.53	0.593	−0.053	
**Emotional exhaustion**						
Work engagement	−6.411	0.832	−7.71	0.000	–0.571*	**3**
Workplace incivility	No path					
Quality of work-life	No path					
Perceived organizational support	−2.624	0.872	−3.01	0.003	–0.226*	**5**
**Depersonalization**						
Work engagement	−1.845	0.576	−3.21	0.001	–0.292*	**4**
Workplace incivility	No path					
Quality of work-life	No path					
Perceived organizational support	−1.329	0.603	−2.20	0.028	–0.204*	
**Personal accomplishment**						
Work engagement	5.065	0.525	9.66	0.000	0.683*	**1**
Workplace incivility	No path					
Quality of work-life	No path					
Perceived organizational support	−0.261	0.608	−0.43	0.668	−0.034	

*Numbers that are bold also indicate top ranked numbers (just to make them stand out)*.

##### Mediating Role of Work Engagement

Quality of Work-life impacted each of the relationships between the organizational factors and all three subcomponents of Burnout through the mediation of Work Engagement. Quality of Work-life has a statistically significant indirect effect on Emotional Exhaustion through Work Engagement of β = −0.332, *z* = −4.47, *p* < 0.001. Quality of Work-life has a statistically significant indirect effect on Depersonalization through Work Engagement of β = −0.170, *z* = −2.77, *p* = 0.006. Quality of Work-life has a statistically significant indirect effect on Personal Accomplishment through Work Engagement of β = 0.397, *z* = 4.73 *p* < 0.001. Workplace Incivility and Perceived Organizational Support did not have any statistically significant indirect effect on the subcomponents of Burnout mediated by Work Engagement (Table 7).

**Table 7:** Indirect effects with standardized coefficients.

	**Coefficient**	**Standard Error (S.E.)**	***z***	***P* > |*z*|**	**Standardized coefficient (β)**	**Rank**
**Work engagement**						
Workplace incivility	No path					
Quality of work-life	No path					
Perceived organizational support	No path					
**Emotional exhaustion**						
Work engagement	No path					
Workplace incivility	−0.610	0.635	−0.96	0.336	−0.051	
Quality of work-life	−3.901	0.873	−4.47	0.000	–0.333*	**2**
Perceived organizational support	0.353	0.663	0.53	0.594	0.030	
**Depersonalization**						
Work engagement	No path					
Workplace incivility	−0.176	0.189	−0.93	0.354	−0.026	
Quality of work-life	−1.122	0.406	−2.77	0.006	–0.170*	**3**
Perceived organizational support	0.102	0.193	0.53	0.599	0.016	
**personal accomplishment**						
Work engagement	No path					
Workplace incivility	0.482	0.500	0.96	0.335	0.061	
Quality of work-life	3.082	0.651	4.73	0.000	0.397*	**1**
Perceived organizational support	−0.279	0.524	−0.53	0.595	−0.036	

*Numbers that are bold also indicate top ranked numbers (just to make them stand out)*.

#### Objective 2: Ranking of Variables

Based on the net value of the standardized coefficients representing the total effects, the strength of the relationships amongst the variables included in the path analysis rank in the following order from strongest to weakest: (1) Work Engagement and Personal Accomplishment, (2) Quality of Work-life and Work Engagement, (3) Work Engagement and Emotional Exhaustion, (4) Quality of Work-life and Personal Accomplishment, (5) Quality of Work-life and Emotional Exhaustion, and (6) Perceived Organizational Support and Emotional Exhaustion (Table 8).

**Table 8:** Total effects with standardized coefficients.

	**Coefficient**	**Standard error (S.E.)**	***z***	***P* > |*z*|**	**Standardized coefficient (β)**	**Rank**
**Work engagement**						
Workplace incivility	0.095	0.098	0.97	0.333	0.090	
Quality of work-life	0.608	0.111	5.48	0.000	0.582^*^	**2**
Perceived organizational support	−0.0550	0.103	−0.53	0.593	−0.053	
**Emotional exhaustion**						
Work engagement	−6.411	0.832	−7.71	0.000	–0.571^*^	**3**
Workplace incivility	−0.6102	0.635	−0.96	0.336	−0.051	
Quality of work-life	−3.901	0.872	−4.47	0.000	–0.332^*^	**5**
Perceived organizational support	−2.271	1.101	−2.06	0.039	–0.196^*^	**6**
**Depersonalization**						
Work engagement	−1.845	0.576	−3.21	0.001	−0.292	
Workplace incivility	−0.176	0.189	−0.93	0.354	−0.026	
Quality of work-life	−1.122	0.406	−2.77	0.006	−0.170	
Perceived organizational support	−1.227	0.644	−1.91	0.057	−0.188	
**Personal accomplishment**						
Work engagement	5.065	0.525	9.66	0.000	0.683^*^	**1**
Workplace incivility	0.482	0.500	0.96	0.335	0.061	
Quality of work-life	3.082	0.651	4.73	0.000	0.397^*^	**4**
Perceived organizational support	−0.540	0.855	−0.63	0.528	−0.070	

* *denotes statistical significance at p < 0.001. Numbers that are bold also indicate top ranked numbers (just to make them stand out)*.

## Discussion

We tested a model of the relationships amongst modifiable environmental and attitudinal factors and Burnout, and ranked the strength of the relationship in order to guide managers and leaders on how to better support nursing staff. Our model had good fit, supporting the hypothesized relationships between the work environment, work attitudes and work outcomes assessed.

### Direct Effects of Work Environment on Burnout

We observed a significant positive relationship between Quality of Work-life and Work Engagement, a relationship that has been supported in previous work on registered nurses (44). By addressing elements of work-life such as physical needs (e.g., compensation, time off, health benefits) and esteem and actualization needs (e.g., relationships, skill development, and the realization of one's potential) organizations can directly impact Work Engagement. Not only does this improve the well-being of clinicians, but their enhanced well-being has also been shown to improve patient care as well as increase hospital revenues (45). This is also in congruence with the Job-Demands Resources model (JD-R) that states greater job demands (stress) and lack of resources (defined as factors similar to those of Quality of Work-life) results in greater Burnout and the inverse results in greater Work Engagement (46).

Additionally, we found that Work Engagement has significant negative/inverse relationships with all of the sub-components of Burnout; a result that is also consistent with the results presented by Hetzel-Riggin et al. in 2020 when evaluating nurses and nursing students (47). By improving Work Engagement, organizations can significantly influence the experience of Emotional Exhaustion, Depersonalization, and Personal Accomplishment in their staff. However, directly modifying work attitudes, and more specifically Work Engagement, is challenging (14). The mediating role of Work Engagement between the work environment and Burnout that is identified in this study and explained below.

### Mediating Role of Work Engagement

We identified Work Engagement as a significant mediator of the effect of Quality of Work-life on the subcomponents of Burnout. These results illuminate an important point: intervening on the work environment, without considering the mediating effects of Work Engagement, may have a limited effect on Burnout. Berta et al.'s study on Health Support Workers supports our model by where features of the work environment are related to Burnout through work attitudes, such as Work Engagement (14). Addressing Quality of Work-life occurs at the interface of the work environment and individuals' role identities. Some strategies to address Quality of Work-life include decentralized organizational structures, improved team work, key stakeholder involvement in decision-making, performance feedback and role clarity, incentive plans, and promotion opportunities from within (48, 49). By improving work-life, there is also an opportunity to improve employees' overall life, through the concept of spillover (49). Sirgy et al. explain that spillover occurs when our reactions to work-life spill over into our non-work life, and note that the reverse can also occur (48). These could provide strategies for organizational leaders to influence pediatric nurse Burnout through Work Engagement with the modulation of the work environment.

### Influencing Pediatric Critical Care Nurse Burnout

All three subcomponents of Burnout were influenced by Work Engagement. This means that hospital leadership can address Burnout *through* the influence of Quality of Work-life on Work Engagement.

Importantly, these results provide an evidence-based, directed strategy for administrators to target in a resource-limited system. The more engaged the nurse is with their work, the greater their sense of more Personal Accomplishment, and the less Emotional Exhaustion and feeling of Depersonalization (cynicism) they experience. This is supported by previous literature on the impact of Work Engagement on Burnout (14, 50). Work Engagement can mediate the relationship between the demands of the job and nurse Burnout (47, 51, 52). Nurse Work Engagement also impacts the patients' experience of care (53). Increased nurse Work Engagement has been shown to have positive effects on both personal and organizational outcomes. To be able to increase nurses' engagement with their patients and families we must think about possible interventions in the same light. Highly engaged nurses are essential for ethical, safe, and comprehensive care (34, 50). As Work Engagement is a work attitude that is difficult to directly influence, addressing areas of the work environment are instrumental in improving Work Engagement and, subsequently, Burnout. Quality of Work-life is not only directly correlated with Work Engagement, it is influenced by an employee's satisfaction with how their needs are being met through the resources, outcomes and activities that are derived from their participation in work, indicating that improving these factors of the work environment will also have a positive impact on nurse Burnout (26).

This study illustrates the importance of the impact of the work environment on Work Engagement and, subsequently, Burnout. We are hopeful that this data, and studies like it, will reinforce the thinking that workplace interventions can contribute in a meaningful way to reducing nurse Burnout. Many current workplace well-being recommendations focus only on self-care for pediatric nurse Burnout—our findings highlight that this recommendation is incomplete, and there are ways leadership can adapt the work environment to also optimize well-being (54). More needs to be done at an organizational level to intervene on the factors that significantly impact pediatric nurse Burnout in the workplace, as demonstrated in this study.

### Limitations

This is a single center study in a Western setting, thus local context and experience limits generalizability (55). This is a cross-sectional study with a modest response rate which limits causal and temporal inference. Nurses historically have fairly poor survey response rates (<60%) (56). The results are sufficient to provide targeted recommendations for interventions at this study site (57), and, by providing a detailed description of the study context, the findings aim to be reproducible and adaptable to other health care settings and populations such as other pediatric critical care units and even pediatric nurses as a whole. The data reflects nurses who chose to participate in the study and may be influenced by selection bias. Effort was made to recruit a sample that is representative of the critical care nursing population at SickKids through distribution to all eligible participants, however despite our best efforts, the sample is not identical to the actual sample distribution. There is also vulnerability to possible bias in the responses due to perceived social desirability, despite anonymity. Participants self-selected to participate in the study; this could have introduced bias in that those with the most extreme feelings may be over-represented.

Path analysis is an explanatory technique and thus is guided by known or hypothesized relationships from the literature. It is important to note that the primary limitation of path analysis is that it does not infer causality or directionality (58).

We acknowledge the impact of the COVID-19 global pandemic and, specifically, its impact on front line essential workers such as pediatric critical care nurses. Nurses, now more than ever, are experiencing the impacts of their work on their well-being; these results will be timely and readily implementable. Further research to confirm and explore these results with pediatric critical care nurses is needed to fully illuminate the conclusions and to design practical interventions to address Burnout. Phase 2 of this study will aim to address this component.

### Theoretical Contributions

At this time, and to our knowledge, there are no previous studies that have considered all of the concepts explored here simultaneously, nor could we find previous studies that have ranked the correlation of work attitudes and work environment factors' contributions to pediatric nurse Burnout. Therefore, the findings of this study advance the understanding of the impacts of the work environment and work attitudes on the work outcome of Burnout in pediatric critical care nurses.

## Conclusion

We found that, in this single center study of pediatric critical care nurses, Burnout levels were high. Pediatric critical care nurse Burnout was most impacted by Work Engagement and quality of work life. Work Engagement is a significant mediator between the work environment and the subcomponents of Burnout. Future interventions for pediatric nurse Burnout by modifying work environment, particularly through the modulation of Work Engagement, have the potential to positively impact the well-being of nurses, and ultimately the care they provide to our most vulnerable patients.

## Data Availability Statement

The raw data supporting the conclusions of this article will be made available by the authors, without undue reservation.

## Ethics Statement

This study was approved by the Research Ethics Board at the Hospital for Sick Children in Toronto, Canada (REB #1000072502). It is also approved by the University of Toronto. The patients/participants provided their written informed consent to participate in this study.

## Author Contributions

LB was involved in the study design, data collection, data analysis, data interpretation, and drafting and finalizing the manuscript. WB and KC were involved in data interpretation, and substantively revised the manuscript for important intellectual content. KW was involved in the study design, data interpretation, and substantively revised the manuscript for important intellectual content. All authors read and approved the final manuscript and agree both to be personally accountable for their own contributions and to ensure that questions related to the accuracy or integrity of any part of the work are appropriately investigated, resolved, and the resolution documented in the literature.

## Funding

This study is funded by the Alma Rae Nursing Scholarship and through the Lawrence S. Bloomberg Faculty of Nursing Doctoral Program and the Grace Evelyn Simpson Reeves Award through the Hospital for Sick Children (Toronto, Canada).

## Conflict of Interest

The authors declare that the research was conducted in the absence of any commercial or financial relationships that could be construed as a potential conflict of interest.

## Publisher's Note

All claims expressed in this article are solely those of the authors and do not necessarily represent those of their affiliated organizations, or those of the publisher, the editors and the reviewers. Any product that may be evaluated in this article, or claim that may be made by its manufacturer, is not guaranteed or endorsed by the publisher.

## Abbreviations

PICU: Pediatric Intensive Care Unit
CCCU: Cardiac Critical Care Unit
NICU: Neonatal Intensive Care Unit
MBI: Maslach Burnout Inventory
POS: Perceived Organizational Support
QWL: Quality of Work-life
WE: Work Engagement
EE: Emotional Exhaustion
DP: Depersonalization
PA: Personal Accomplishment.
